# Sex-specific differences in systemic immune responses in MIS-C children

**DOI:** 10.1038/s41598-024-52116-1

**Published:** 2024-01-19

**Authors:** Anuradha Rajamanickam, Nathella Pavan Kumar, Aishwarya Venkataraman, Poovazhagi Varadarjan, Elilarasi Selladurai, Thangavelu Sankaralingam, Kannan Thiruvengadam, Ramya Selvam, Akshith Thimmaiah, Suresh Natarajan, Ganesh Ramaswamy, Sulochana Putlibai, Kalaimaran Sadasivam, Balasubramanian Sundaram, Syed Hissar, Uma Devi Ranganathan, Subash Babu

**Affiliations:** 1 National Institutes of Health-National Institute for Research in Tuberculosis – International Center for Excellence in Research, Chennai, India; 2 National Institute for Research in Tuberculosis, Chennai, India; 3 Institute of Child Health and Hospital for Children, Chennai, India; 4 Dr. Mehta’s Children’s Hospital, Chennai, India; 5 Rainbow Children’s Hospital, Chennai, India; 6 Kanchi Kamakoti CHILDS Trust Hospital, Chennai, India; 7 Laboratory of Parasitic Diseases, National Institute of Allergy and Infectious Diseases, National Institutes of Health, Bethesda, MD USA

**Keywords:** Immunology, Chemokines, Complement cascade, Cytokines

## Abstract

Multisystem Inflammatory Syndrome in Children (MIS-C) is a rare manifestation of Severe Acute Respiratory Syndrome-CoronaVirus-2 (SARS-CoV-2) infection that can result in increased morbidity and mortality. Mounting evidence describes sex disparities in the clinical outcomes of coronavirus disease 2019 (COVID-19). However, there is a lack of information on sex-specific differences in immune responses in MIS-C. This study is an observational and cross-sectional study and we wanted to examine immune parameters such as cytokines, chemokines, acute phase proteins (APPs), growth factors, microbial translocation markers (MTMs), complement components and matrix metalloproteinases (MMPs) in MIS-C children, based on sex. Male children were associated with heightened levels of pro-inflammatory cytokines—IFNγ, IL-2, TNFα, IL-1α, IL-1β, IL-6, IL-12, G-CSF and GM-CSF, chemokines-CCL2, CCL11, CXCL1, CXCL8 and CXCL10, acute phase proteins-α-2M, CRP, growth factors VEGF and TGFα, microbial translocation markers- iFABP, LBP, EndoCAb, complement components—C1q, MBL and C3 and matrix metalloproteinases MMP-8 and MMP-9 compared to female children with MIS-C. These results indicate that the heightened immune response in males is a characteristic feature of MIS-C. These findings might explain the differential disease pathogenesis in males compared to females with MIS-C and facilitate a deeper understanding of this disease.

## Introduction

Multisystem Inflammatory Syndrome in Children (MIS-C) is a hyperinflammatory sequelae of Severe Acute Respiratory Syndrome-CoronaVirus-2 (SARS-CoV-2) infection in children^1–3^. Several children present with severe multi-organ dysfunction and an exaggerated inflammatory response as a late manifestation of SARS-CoV-2 infection (within 4–6 weeks)^1^. Previous studies have described exaggerated inflammatory responses that are present in MIS-C including elevated levels of pro-inflammatory cytokines, chemokines, growth factors, complement activation, acute phase proteins, activated neutrophils and monocytes, thrombocytopenia, and lymphopenia^4^. It is widely known that biological sex could have an impact on the probability and susceptibility to disease and its sequelae^5^ and between-sex differences have been recognized in medical research. In this context, in previous epidemics of COVID (Acute Respiratory Syndrome-CoronaVirus (SARS-CoV), the Middle East Respiratory Syndrome-CoronaVirus (MERS-CoV)), studies determined that sex differences have a different impact on disease severity and clinical outcomes^6,7^.

Various epidemiologic reports have demonstrated the presence of sex disparities in COVID-19 outcomes^8^. Several studies have shown that men were more frequently affected by COVID-19 than women^6,9^ and sex has also been recognized as a determinant of the progression and health outcomes of COVID-19^10^. Various studies reported that males may be highly represented in MIS-C, however, only a minor male dominance is observed in six studies^3,9,11–17^. As of November 27, 2023, there have been 9,604 reported cases of MIS-C in the United States, with 79 reported deaths. The median age of affected cases is 9 years, and notably, 60% of the reported MIS-C cases involve male children. These figures are based on data from the Centers for Disease Control and Prevention (CDC) COVID Data Tracker, accessed on December 8, 2023 (https://covid.cdc.gov/covid-data-tracker/#mis-national-surveillance)^18^.

To delineate the immune responses in males and females with MIS-C, we performed a detailed analysis of the sex differences in immune responses by assessing the levels of plasma cytokines, chemokines, acute phase proteins, growth factors, microbial translocation markers, complement proteins and matrix metalloproteinases. Our results indicate that the heightened immune responses in males lead to an exaggerated inflammatory response which could potentially predispose male children to hyperinflammation and poor innate and adaptive immune responses.

## Results

### Basic characteristics

We measured an array of immunological parameters on stored plasma samples of 65 MIS-C children hospitalized from June to September 2020. As shown in Tables 1 and 2, the median age was 5.8 years (range 1 month to 14 years), 54% (35/65) were males and 46% were females. All MIS-C children were seropositive (IgG) among whom 29% of males and 23% of females had a severe disease that required PICU care. Among the laboratory parameters, WBC, and lymphocyte levels were significantly increased in males compared with females in MIS-C children. Among the biochemical parameters analysed, the levels of CRP, ferritin and Troponin-I levels were significantly higher in males when compared with female MIS-C children.

**Table 1:** MIS-C children's demographic and biochemical parameters.

Parameters	Male	Female	*p* value
	n = 35	n = 30	
Age in median (month–years)	5.8 (1–12)	5.9 (1–14)	0.8975
Weight, (Kg)	9.2 (3.2–24)	21.3 (6.0–42)	< 0.0001
Height, (cm)	83.47 (43–112)	123.1 (100–142)	0.0001
BMI, (kg/m^2^)	19.2 (13.6–22.8)	20.3 (13.7–21.9)	0.7385
CRP median (IQR) (< 3 mg/L)	101 (3.5–473)	93 (3.5–313)	0.9857
WBC 10^3^ cells/ul median /range	4.3 (2.77–8.870)	5.6 (3.010–8.285)	0.0121
Hb, (g/dl) median /range	11.05 (9.20–14.65)	11.00 (7.87–12.11)	0.7976
Lymphocyte, (/mm^3^) (1500–4000) median (IQR)	1343 (330–6270)	2895 (450–7280)	0.5438
Neutrophils, (/mm^3^) (1500–7000) median (IQR)	11,179 (8500–15,900)	10,032 (5500–11,200)	0.6132
Platelets, (200–450) × 10^9^/L median (IQR)	107 (86–255)	147 (58–255)	0.4974
CRP, median (IQR) (< 3 mg/L)	101 (3.5–473)	93 (3.5–313)	0.0121
Sodium, (135–145 mmol/l) median (IQR)	133 (124–139)	142 (132–138)	0.2145
Ferritin (ng/mL) (7–140) median (IQR)	1348 (321–5377)	1148 (306–4738)	0.0334
Potassium 3.70–5.20 (millimol/L)	4.3 (2.5–6.3)	4.2 (3–47.9)	0.3630
Creatinine, mg/dL	0.41 (0.1—4.6)	0.57 (0.1–9.7)	< 0.0001
Pro-BNP, pg/mL	2889.5 (465–23,157)	24,936 (64–25,000)	0.2280
Troponin-I, pg/mL	6.1 (1.5–16.30)	5.4 (1.3–14.46)	0.0238

**Table 2:** MIS-C children’s clinical parameters of the study population.

	Male	Female
Age median (years, IQR)	5.8 (1–14)	5.9 (1–14)
Gender n (%)	35 (54%)	30 (46%)
RT-PCR positive n (%)	0	0
Serology IgG positive n (%)	35 (100%)	30 (100%)
Underlying conditions n (%)	3 (4.5%)	1 (1.5%)
Co-existing infections n (%)	4 (6%)	1 (1.5%)
Median duration since proven or suspected COVID illness or contact (weeks, range)	3 w (10 d–4 w)	3 w (10 d–4 w)
COVID-19 symptoms n (%)		
Fever	35 (100%)	30 (100%)
Gastrointestinal	28 (80%)	24 (80%)
Respiratory	8 (23%)	7 (23%)
Mucocutaneous	27 (77%)	22 (73%)
Asymptomatic	0	0
Cardiovascular symptoms/signs		
Hypotension	20 (57%)	14 (47%)
Shock	1 (2.9%)	2 (6%)
Coronary artery dilatation	3 (9%)	2 (7%)
Myocardial dysfunction	22 (63%)	12 (40%)
Median duration of stay	5 (3–18)	4 (1–15)
Treatment n (%)		
IVIG	25 (71%)	19 (63%)
Steroids	24 (69%)	23 (77%)
PICU admission	19 (54%)	15 (50%)
Antibiotics	34 (97%)	26 (87%)
Tocilizumab (8 mg/kg)	2 (6%)	1 (3%)
Respiratory support n (%)		
Mechanical ventilation	1 (3%)	0
HHFNC	3 (9%)	2 (7%)
Oxygen	5 (14%)	4 (13%)
Cardiovascular support n (%)		
Inotropes	20 (57%)	14 (47%)
Fluid bolus	23 (66%)	19 (63%)

### Heightened plasma levels of cytokines are associated with male MIS-C children

**Figure 1:**
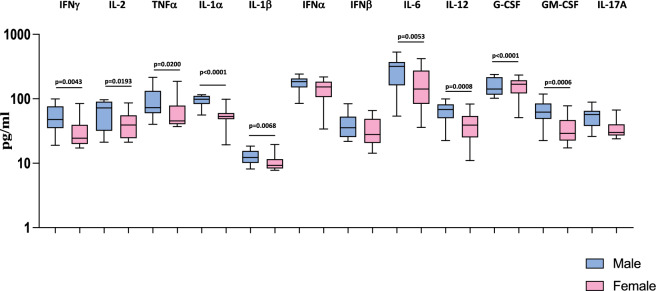
Heightened plasma levels of cytokines are associated with male MIS-C children. The plasma levels of IFNγ, IL-2, TNFα, IL-1α, IL-1β, IL-6, IL-12, G-CSF, GM-CSF and IL-17A were measured in male MIS-C (n = 35), and female MIS-C children (n = 30). The data are represented as Box and Whiskers plots. p values were calculated using the Mann–Whitney U test.

We wanted to examine the influence of sex on the levels of cytokine responses in MIS-C children. Therefore, we measured the levels of cytokines (IFNγ, IL-2, TNFα, IL-1α, IL-1β, IFNα, IFNβ, IL-6, IL-12, G-CSF, GM-CSF and IL-17A) in male and female MIS-C children. As shown in Fig. 1, we observed that male MIS-C children exhibited significantly increased levels of IFNγ (GM of 47.24 pg/ml in males compared to 29.44 pg/ml in females; *p* = 0.0043), IL-2 (GM of 54.92 pg/ml in males compared to 38.19 pg/ml in females; *p* = 0.0193), TNFα (GM of 80.25 pg/ml in males compared to 56.93 pg/ml in females; *p* = 0.0200), IL-1α (GM of 93.67 pg/ml in males compared to 52.45 pg/ml in females; *p* < 0.0001), IL-1β (GM of 12.56 pg/ml in males compared to 10.19 pg/ml in females; *p* = 0.0068), IL-6 (GM of 251.8 pg/ml in males compared to 138 pg/ml in females; *p* = 0.0053), IL-12 (GM of 60.09 pg/ml in males compared to 35.72 pg/ml in females; *p* = 0.0008), G-CSF (GM of 61.35 pg/ml in males compared to 32.77 pg/ml in females; *p* < 0.0001) and GM-CSF (GM of 49.53 pg/ml in males compared to 33.56 pg/ml in females; *p* = 0.0006) compared to female MIS-C children. Thus, male MIS-C children have exacerbated pro-inflammatory cytokine responses compared to female MIS-C children.

### Heightened plasma levels of CC and CXC chemokines are associated with male MIS-C children

**Figure 2:**
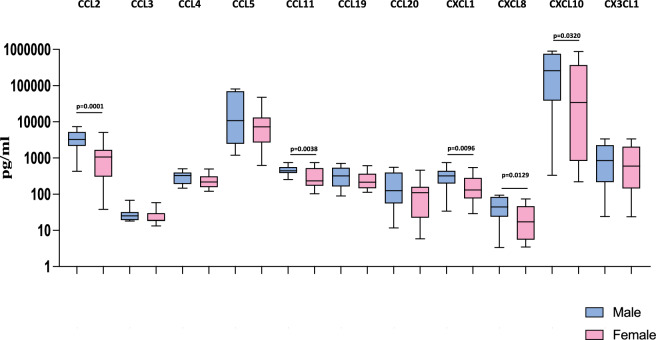
Heightened plasma levels of CC and CXC chemokines are associated with male MIS-C children. The plasma levels of CCL2, CCL3, CCL4, CCL5, CCL11, CCL19, CCL20, CXCL1, CXCL8, CXCL10 and CX3CL1 were measured in male MIS-C (n = 35), and female MIS-C children (n = 30). The data are represented as Box and Whiskers plots. *p* values were calculated using the Mann–Whitney U test.

Next, we wanted to examine the influence of sex on the levels of chemokines in MIS-C children. Therefore, we measured the levels of chemokines (CCL2, CCL3, CCL4, CCL5, CCL11, CCL19, CCL20, CXCL1, CXCL8, CXCL10 and CX3CL1) in male and female MIS-C children. As shown in Fig. 2, we observed that male MIS-C children exhibited significantly increased levels of CCL2 (GM of 2952 pg/ml in males compared to 792.3 pg/ml in females; *p* = 0.0001), CCL11 (GM of 453.9 pg/ml in males compared to 274.5 pg/ml in females; *p* = 0.0038), CXCL10 (GM of 280 pg/ml in males compared to 140.1 pg/ml in females; *p* = 0.0096), and CXCL10 (GM of 129,033 pg/ml in males compared to 19,960 pg/ml in females; *p* = 0.0320) compared to female MIS-C children. Thus, male MIS-C children have exacerbated chemokine responses compared to female MIS-C children.

### Heightened plasma levels of acute phase proteins, growth factors and microbial translocation markers are associated with male MIS-C children

**Figure 3:**
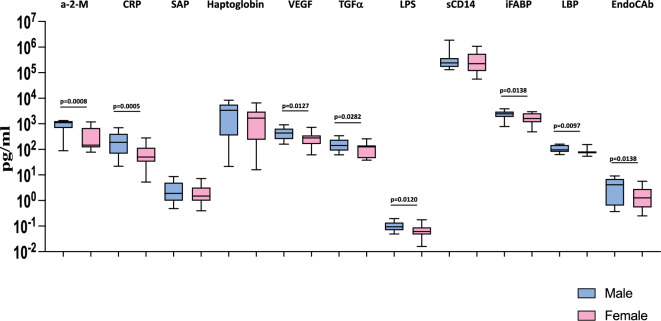
Heightened plasma levels of acute phase proteins, growth factors and microbial translocation markers are associated with male MIS-C children. The plasma levels of C-reactive protein (CRP), alpha-2 macroglobulin (α-2 M), haptoglobin, Serum Amyloid P (SAP), VEGF, TGFα, LPS, lipid-binding protein (LBP), endotoxin core antibodies IgG (EndoCAb), intestinal fatty acid binding protein (iFABP) and sCD14 were measured in male MIS-C (n = 35), and female MIS-C children (n = 30). The data are represented as Box and Whiskers plots. *p* values were calculated using the Mann–Whitney U test.

Further, we wanted to examine the influence of sex on the levels of acute phase proteins and microbial translocation markers in MIS-C children. Therefore, we measured the levels of acute phase proteins α-2-M, CRP, SAP, and Hp, growth factors (VEGF and TGFα) and microbial translocation markers (LPS, sCD14, iFABP, LBP and EndoCAb) in male and female MIS-C children. As shown in Fig. 3, we observed that male MIS-C children exhibited significantly elevated levels of α-2-M (GM of 716.8 pg/ml in males compared to 254.1 pg/ml in females; *p* = 0.0008) and CRP (GM of 174.6 pg/ml in males compared to 52.43 pg/ml in females; *p* = 0.0005), growth factors VEGF (GM of 417.4 pg/ml in males compared to 248.3 pg/ml in females; *p* = 0.0127), TGFα (GM of 149.6 pg/ml in males compared to 92.47 pg/ml in females; *p* = 0.0282) and microbial translocation markers, LPS (GM of 0.09817 pg/ml in males compared to 0.06585 pg/ml in females; *p* = 0.0120), iFABP (GM of 2259 pg/ml in males compared to 1574 pg/ml in females; *p* = 0.0138), LBP (GM of 105.2 pg/ml in males compared to 81.04 pg/ml in females; *p* = 0.0097), and EndoCAb (GM of 2.694 pg/ml in males compared to 1.232 pg/ml in females; *p* = 0.0127) compared to female MIS-C children. Thus, male MIS-C children have exacerbated acute phase protein, growth factor and microbial translocation marker responses compared to female MIS-C children.

### Heightened plasma levels of complement components and complement regulators are associated with male MIS-C children

**Figure 4:**
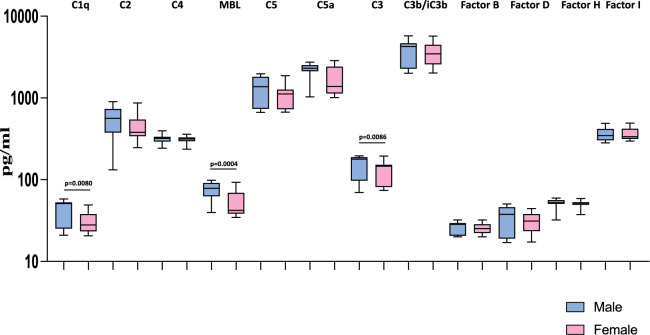
Heightened plasma levels of complement components and complement regulators are associated with male MIS-C children. The plasma levels of complement components (C1q, C2, C3, C4, C4b, MBL, C5), regulators (factor B, factor D, factor H and factor I) and activation products (iC3b, C5b) were measured in male MIS-C (n = 35), and female MIS-C children (n = 30). The data are represented as Box and Whiskers plots. *p* values were calculated using the Mann–Whitney U test.

Next, we wanted to examine the influence of sex on the levels of complement components and complement regulators in MIS-C children. Therefore, we measured the levels of complement proteins like C1q, C2, C3, C3b/iC3b, C4, C4b, C5, C5a, MBL and complement regulatory proteins like Factor B, Factor D, Factor H and Factor I in male and female MIS-C children. As shown in Fig. 4, we observed that male MIS-C children exhibited significantly elevated levels of C1q (GM of 31.89 pg/ml in males compared to 29.33 pg/ml in females; *p* = 0.0080), MBL (GM of 71.93 pg/ml in males compared to 49.24 pg/ml in females; *p* = 0.0004), and C3 (GM of 145 pg/ml in males compared to 118.9 pg/ml in females; *p* = 0.0086) compared to female MIS-C children. Thus, male MIS-C children have exacerbated levels of complement components and complement regulators compared to female MIS-C children.

### Heightened plasma levels of matrix metalloproteinases are associated with male MIS-C children

**Figure 5:**
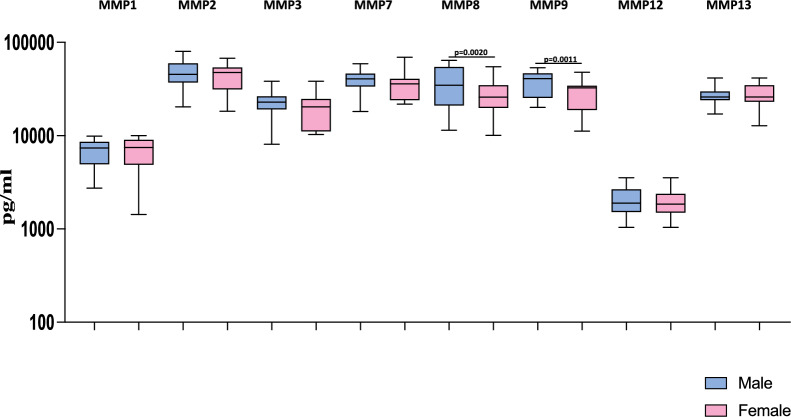
Heightened plasma levels of matrix metalloproteinases are associated with male MIS-C children. The plasma levels of Matrix Metallo Proteinases (MMP) MMP1, MMP2, MMP3, MMP7, MMP8, MMP9, MMP12 and MMP13 were measured in male MIS-C (n = 35), and female MIS-C children (n = 30). The data are represented as Box and Whiskers plots with each circle representing a single individual. *p* values were calculated using the Mann–Whitney U test.

Next, we wanted to examine the influence of sex on the levels of matrix metalloproteinases in MIS-C children. Therefore, we measured the levels of matrix metalloproteinases like MMP1, MMP2, MMP3, MMP7, MMP8, MMP9, MMP12, and MMP13 in male and female MIS-C children. As shown in Fig. 5, we observed that male MIS-C children exhibited significantly increased levels of MMP8 (GM of 32,472 pg/ml in males compared to 24,870 pg/ml in females; *p* = 0.0004) and MMP9 (GM of 35,921 pg/ml in males compared to 26,836 pg/ml in females; *p* = 0.0011) compared to female MIS-C children. Thus, male MIS-C children have exacerbated levels of matrix metalloproteinases compared to female MIS-C children.

### Immune parameters can strongly discriminate sex-based immune responses in MIS-C children

**Figure 6:**
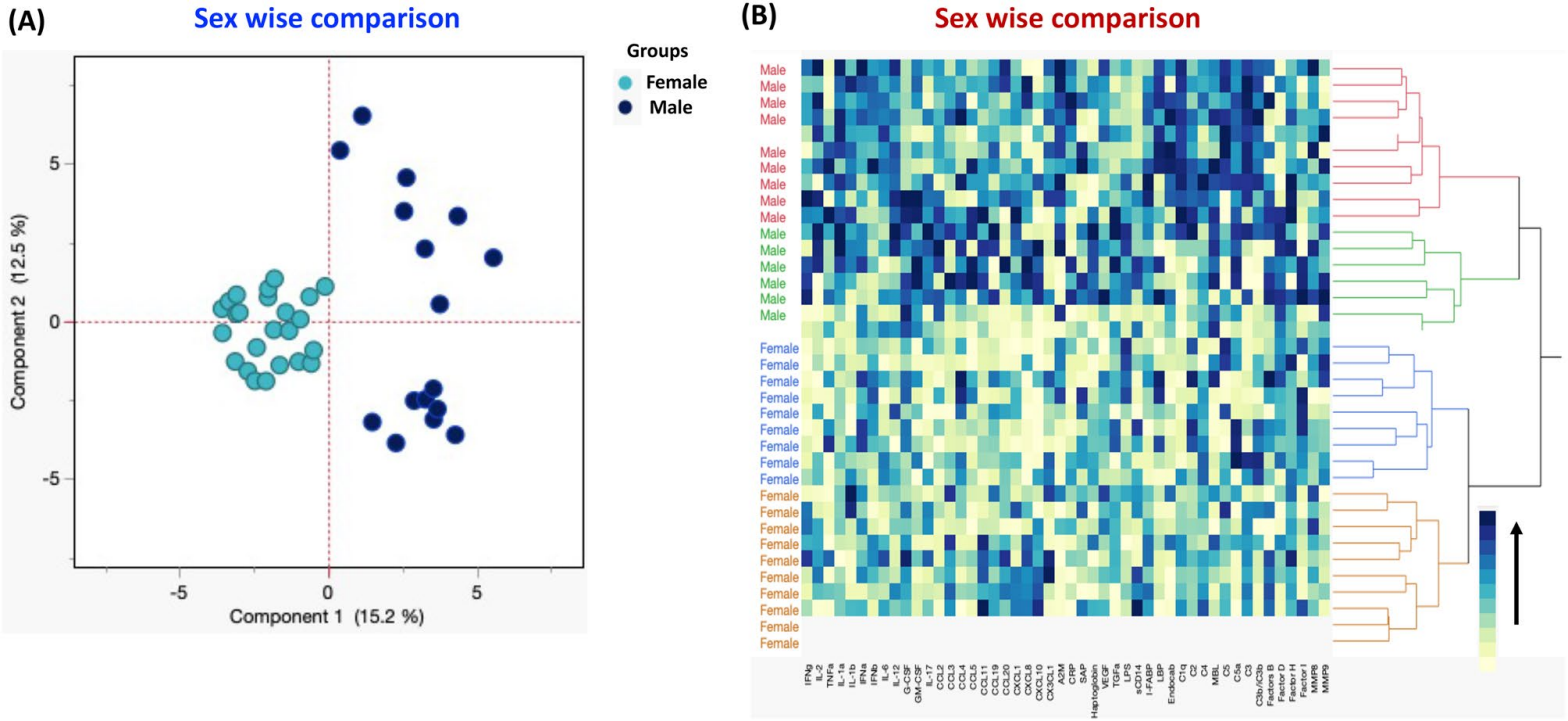
Immune parameters can strongly discriminate sex-based immune responses in MIS-C children. (**A**) PCA (Principal component analysis) plot computing normalized immunological parameters after excluding those factors with commonalities as low as 0.5 we used Groups IFNγ, IL-2, TNFα, IL-1α, IL-1β, IFNα, IFNβ, IL-6, IL-12, G-CSF, GM-CSF, IL-17, CCL2, CCL3, CCL4, CCL5, CCL11, CCL19, CCL20, CXCL1, CXCL8, CXCL10, CX3CL1, A2M, CRP, SAP, Haptoglobin, VEGF, TGFα, LPS, sCD14, I-FABP, LBP, EndoCAb, C1q, C2, C4, MBL, C5, C5a, C3, C3b/iC3b, Factors B, Factor D, Factor H and Factor I, MMP8 and MMP9 in the combination of male and female experimental groups male MIS-C (Coloured in maroon) vs female MIS-C (Coloured in mustard). The PCA shows the two principal components of variation, accounting for 15.7% (x-axis) and 12.5% (y-axis). (**B**) Cytokines, chemokines, acute phase proteins, growth factors, microbial translocation markers, complement components and matric metalloproteinases are depicted according to the score denoted in the colour-scale bar. Associated horizontal dendrograms denote the patients’ clustering. The colour scale yellow colour depicts lower expression and dark blue colour depicts higher expression.

Using immunological parameters like cytokines, chemokines, acute-phase proteins, growth factors, microbial translocation markers, complement components, and MMPs, we performed PCA (principal component analysis) plot computing normalised immune markers after excluding those factors with commonalities as low as 0.5 (Fig. 6A) in order to determine the discriminatory ability of immune parameters between male and female of MIS-C children. Additionally, we used data sets from two groups of kids to do hierarchical clustering analysis utilising cytokines, chemokines, acute-phase proteins, growth factors, microbial translocation indicators, complement components, and MMPs. For cellular subgroups, a heatmap and dendrogram are shown (Fig. 6B). Ward's supervised clustering algorithm and index were used to create the dendrogram, which is demonstrated to be capable of discrimination.

## Discussion

In this study, we aimed to determine the influence of sex on immune responses in MIS-C children. We observed that male children with MIS-C exhibited significantly elevated levels of pro-inflammatory cytokines, chemokines, growth factors, microbial translocation markers (MTMs) and Matrix Metalloproteinases (MMPs) when compared with female MIS-C children. Increasing evidence that the male sex is a risk factor for COVID-19 mortality and more severe illness. Our data indicate significant disparities in the immune responses in male and female children with MIS-C and suggest a possible immunological underpinning of the different means of disease progression among sexes. These data also offer a possible basis for taking sex-dependent methods for prognosis, prevention, care, and therapy for the patient with MIS-C.

It has been established that the immune system differs across the sexes and is varied in terms of both innate and adaptive immunity^19^. Biological sex has an impact on innate and adaptive immune responses to self and foreign antigens, resulting in sex disparities in autoimmunity, and responses to infections and vaccines^20^. The activity of X-linked genes can be used to explain the discrepancy in the impact of sex-specific diseases. There are at least two known variables that affect sexual dimorphism's impact on immunity: hormonal changes and heredity, namely the X chromosome. Some of the immune response-related proteins encoded by genes on the X chromosome include toll-like receptors and interleukins^19^. Additionally, the sex-specific expressions of TMPRSS2 may help to partially explain the COVID-19 pandemic's male preponderance^21^. Some variations can also be attributed to variations in the activity and expression of the human angiotensin-converting enzyme 2 (ACE2), the functional receptor for SARS-CoV-2^9^.

Past studies have shown that cytokines and chemokines were significantly elevated and have a role in the pathogenesis of the MIS-C^3,22,23^. In an adult SARS-CoV-2 study, adult females exhibited higher levels of interferon-α (IFNα) than adult males^24^. On the contrary, a study revealed that male patients exhibited elevated plasma levels of innate immune cytokines such as IL-8, IL-18, and the chemokine CCL5, along with a pronounced induction of monocytes. In contrast, female patients demonstrated a more robust activation of T cells. The study further observed that a deficient T-cell response was negatively correlated with age and led to a more adverse disease outcome in male patients, with no corresponding impact on female patients^25^. Similarly, we observed that male MIS-C children had significantly elevated plasma levels of cytokines and chemokines compared with females. Sex differences in cytokines and chemokines responses to MIS-C in children warrant further investigation in larger cohorts of children.

In MIS-C and COVID-19, CRP levels have been used as a marker of disease pathology^17^. Previously, we have shown that CRP has a major contribution to the pathogenesis of this disease entity in MIS-C and acute COVID-19 children^26^. In this study, we observed male MIS-C children had elevated levels of CRP and a-2-M compared with female MIS-C children, which indicates that male children had higher levels of pathogenesis compared with female children. In the absence of overt bacteremia, microbial translocation is the process through which microbial products like lipopolysaccharide (LPS) and bacterial DNA move from the intestinal lumen to the systemic circulation^27^. Our earlier data determined that MIS-C children had elevated levels of LPS, LBP, and sCD14, all markers of gut damage and microbial translocation across the gastrointestinal tract^26^. Microbial translocation also plays a role in gut microbiome dysbiosis in COVID-19^28^. Recent data revealed that zonulin, a biomarker of intestinal permeability found in children with MIS-C in the GI tract, with subsequent trafficking of SARS-CoV-2 antigens into the bloodstream, leading to hyperinflammation^29^· Myocarditis and/or pericarditis represent infrequent adverse cardiac events observed in adolescent and young adult males following SARS-CoV-2 mRNA vaccination. These events are characterized by acute systemic inflammation, elevated troponin and BNP levels, and abnormalities in cardiac imaging, as documented by Katoto et al.and Anis Barmada et al.^30,31^. Notably, a recent study by Ulucay AS reported a higher susceptibility to myocarditis (both associated with COVID-19 and mRNA vaccines) among males (0.09%) compared to females (0.04%)^32^. In our study, we observed that male children had elevated levels of LPS, iFABP, LBP and EndoCAb compared with female MIS-C children. These results imply that male children could be affected by higher levels of gut damage and microbial translocation across the gastrointestinal tract than female children.

The innate immune system's complement system is an essential component that contributes to the pathogenesis of COVID-19. In an adult healthy Caucasian population study, Alternative Pathway activity, C3, and MBL levels were found to be lower in females compared to males while factor D concentrations were higher^19^. Recently, there have been reports that the dysregulation of complement proteins are involved in the COVID-19 disease pathology in adults and children^33–36^. The immunological characteristics of MIS-C have been shown to include the activation of complement pathways and modification of innate and adaptive immune responses^37^. Previous reports indicated that MIS-C and acute COVID-19 children were associated with highly elevated levels of activation markers of the classical, alternative and terminal pathways^38,39^. Children with SARS-CoV-2 infection exhibited elevated levels of sC5b9, which correlated with disease severity^35,38,40,41^. A recent study with MIS-C children on complement observed elevated levels of activation markers of the classical, alternative and terminal pathways^38^. Our data determine that males have significantly higher complement activity and levels of complement components compared to females. Moreover, our results imply that sex should be considered in complement-associated pathology and also in complement-directed therapies.

Matrix metalloproteinases (MMPs) are important zinc-dependent endo proteases involved in both physiological and pathological ECM remodelling^42^. Increased plasma levels of MMP-9 were found in patients with severe ARDS^43^ and COVID-19 patients^44^. Another study described the immune-based signature of COVID-19 patients, relating serum MMP-9 levels with the severity and as a biomarker of COVID-19^45,46^. It has been observed that in lung tissue from COVID-19 patients, the MMP9 gene is up-regulated, and the protein contributes to cytokine recruitment^47^. We have previously observed that MMPs play a role in the pathogenesis of MIS-C and acute COVID-19 in children^48^. In the current study, we observed male MIS-C children exhibited elevated levels of MMP-8 and MMP-9 which is associated with disease severity and pathogenesis of MIS-C. These results indicate that male children may have more severity and pathology of MIS-C than female children.

Our study has certain limitations, such as the fact that we only looked at children from one location, the limited number of samples, and the lack of longitudinal follow-up data. Our work, on the other hand, breaks new ground by analysing a wide range of immunological measures in both male and female MIS-C children and by offering a more in-depth investigation of inflammatory markers. In conclusion, our work is the first to provide a thorough analysis of sex-based disparities in the immunological parameter profile of Indian children with MIS-C. The fact that sex differences are a significant contributor to individual differences makes understanding them vital. The efficacy of clinical practice, health policy, and treatments will be increased by having a better understanding of the disparities in outcomes between male and female patients. Future studies are necessary to comprehend the reasons for the sex gap, and they may also be of relevance to public health decision-makers.

## Methods

### Study design and participants

We prospectively enrolled children admitted to Kanchi Kamakoti CHILDS Trust Hospital (KKCTH), Chennai, India Institute of Child Health, Dr Mehta’s Children Hospital, Rainbow Children’s Hospital, from 1 June 2020 to 30 September 2020 with MIS-C children. The study population and the enrolment criteria have been previously described^26,48,49^. Briefly, we included children of either sex between 1 month and 14 years of age or whose parents were willing to provide informed consent/assent. Blood collection was performed prior to any immunomodulatory medication. Plasma was isolated and used for measuring multiple immune parameters. The demographic, epidemiological, medical and laboratory data have been previously reported^26,48,49^ and are described in Table 1. Children with MIS-C were diagnosed according to the World Health Organisation (WHO) case definition^50^ and all the enrolled MIS-C cases have no other microbial or viral inflammatory focus. Blood was collected in EDTA tubes (BD Biosciences) or heparin tubes and processed within 4 h of collection at the National Institute for Research in Tuberculosis (NIRT), Chennai. To avoid measurement bias and to increase the precision of the estimates for the accuracy of the assay, the study staff involved in immunological assays were blinded to any clinical data.

### Measurement of cytokines, chemokines and growth factors

Circulating plasma levels of cytokines, chemokines and growth factors were measured by Luminex Magpix Multiplex Assay system (Bio-Rad, Hercules, CA) using Luminex Human Magnetic Assay kit 45 Plex (R & D systems). The lowest detection limits for cytokines were as follows: IFNγ, 5.7 pg/mL; IL-2, 3.6 pg/mL; TNFα, 12.4 pg/mL; IL-1α, 10.6 pg/mL; IL-1β, 3.5 pg/mL; IFNα, 3.9 pg/mL; IFNβ 3.25 pg/mL; IL-6, 9.0 pg/mL; IL-12, 18.5 pg/mL; IL-15, 2.5 pg/mL; IL-17A, 9 pg/mL; IL-3, 17 pg/mL; IL-7, 3.5 pg/mL; G-CSF, 8.4 pg/mL; GM-CSF, 18.4 pg/mL; IL-4, 1.1 pg/mL; IL-5, 6.2 pg/mL; IL-13, 31.8 pg/mL; IL-10, 32.2 pg/mL; IL-25, 18.4 pg/mL; IL-33, 13.8 pg/mL; IL-1Ra, 11.7 pg/mL. The lowest detection limits for chemokines were as follows: CCL2, 5.9 pg/mL; CCL3, 5.1 pg/mL; CCL4, 103.8 pg/mL; CCL5, 297 pg/mL; CCL11, 21.6 pg/mL; CCL19, 3.9 pg/mL; CCL20, 2.4 pg/mL; CXCL1, 19.1 pg/mL; CXCL2, 21.1 pg/mL; CXCL8, 1.4 pg/mL; CXCL10, 2.6 pg/mL and CX3CL1, 188 pg/mL. The lowest detection limits for growth factors were as follows: VEGF, 5.9 pg/mL; EGF, 8.6 pg/mL; FGF-2, 8.7 pg/mL; PDGF-AA, 5.2 pg/mL; PDGF-BB, 7.31 pg/mL; TGFα, 8.6 pg/mL; Flt-3 L, 22.9 pg/mL; Granzyme B (GZB), 4.9 pg/mL; PDL-1, 69.3 pg/mL; TRAIL, 22.5 pg/mL.

### Measurement of acute phase proteins

Plasma levels of alpha-2 macroglobulin (α 2 M), C-reactive protein (CRP), haptoglobin (Hp), and serum amyloid P (SAP), using a Milliplex MAP Human CVD Panel Acute Phase magnetic bead panel 3 from Millipore, were measured using a multiplex platform according to the manufacturer’s instructions. The lowest detection limits for acute phase proteins were as follows: α-2-M, 0.49 ng/mL; CRP, 0.05 ng/mL; Hp, 0.06 ng/mL; and SAP, 0.06 ng/mL.

### Measurement of microbial translocation markers

To inactivate plasma proteins, plasma samples were heated to 75 °C for 5 min. LPS levels were measured using a Limulus amoebocytes lysate assay (Cell Sciences Hycult Biotech, Canton, MA, USA) according to the manufacturer’s protocol. Commercially available enzyme-linked immunosorbent assay (ELISA) kits were used to measure plasma levels of lipid-binding protein (LBP), endotoxin core antibodies IgG (EndoCAb), intestinal fatty acid binding protein (iFABP; all Cell Sciences Hycult Biotech), and sCD14 (R&D Systems, Minneapolis, MN, USA). The lowest detection limits for microbial translocation markers were as follows: LPS, 0.04 EU/mL; LBP, 4.4 ng/mL; EndoCAb, 0.063 GMU/mL; iFABP, 15.6 pg/mL; and sCD14, 250 pg/mL.

### Measurement of complement cascade proteins and complement regulatory proteins

Systemic levels of C1q, C2, C3, C3b/iC3b, C4, C4b, C5, C5a, MBL complement proteins and complement regulatory proteins like Factor B, Factor D, Factor H and Factor I were determined using the Luminex xMAP technology with MILLIPLEX Bead-Based Multiplex Complement Panel I and II Assay kits. The lowest detection limits were as follows: C2: 1.37 ng/mL; C4b: 1.37 ng/mL; C5: 2.74 ng/mL; C5a: 4.12 pg/mL; Adipson/ Complement Factor D: 0.069 ng/mL; Mannose-Binding Lectin (MBL): 0.137 ng/mL; Complement Factor I: 0.69 ng/mL; C1q: 0.08 ng/mL; C3: 0.27 ng/mL; C3b/iC3b: 8.2 ng/mL; C4: 0.55 pg/mL; complement Factor B: 0.08 ng/mL and complement Factor H: 0.041 ng/mL.

### Measurement of matrix metalloproteinases

Circulating plasma levels of MMP-1, MMP-2, MMP-3, MMP-7, MMP-8, MMP-9, MMP-10, MMP-12 and MMP-13 were determined using a multiplex enzyme-linked immunosorbent assay system using the Luminex Magpix Multiplex Assay system; Bio-Rad. MMP levels were measured using a commercially available kit (Luminex Human Magnetic Assay kit 8 Plex from R&D Systems). All the samples were tested in duplicates and averages were used for the analysis. The lowest detection limits were as follows: MMP-1, 115.8 pg/ml; MMP-2, 809 pg/ml; MMP-3, 199.2 pg/ml; MMP-7, 27.7 pg/ml; MMP-8, 31.7 pg/ml; MMP-9, 257.5 pg/ml; MMP-10, 78.4 pg/ml; MMP-12, 18.5 pg/ml; MMP-13, 32.9 pg/ml.

### Statistical analysis

For analysis, MIS-C children were categorised into two groups-males and females. Geometric means (GM) were used for measurements of central tendency. Continuous variables are presented as medians and interquartile ranges (IQRs), and categorical variables are reported as numbers and proportions. Comparison between the groups was performed using the Mann–Whitney U test. *p* ≤ 0.05 was considered statistically significant and all tests were two-sided. Analyses were performed using Graph-Pad PRISM Version 9.0 (GraphPad Software, CA, USA).

### Ethical statement

This study was performed in line with the principles of the Declaration of Helsinki. The Internal Ethics Committee (IEC) of the participating institutes (National Institute for Research in Tuberculosis, Kanchi Kamakoti CHILDS Trust Hospital (KKCTH), Institute of Child Health, Dr Mehta’s Children Hospital, Rainbow Children’s Hospital approved the study. The study was also registered at Clinical Trials Registry India (CTRI/2021/01/030605). The study was also registered with the Clinical Trials registry clinicaltrials.gov (No: NCT04844242). Informed consent was obtained from parents/guardians of all children along with assent where appropriate.

## Data Availability

All data generated or analysed during this study are included in this published article.
